# The co-occurrence of victimization, perpetration, and bystander responses in adolescent school bullying: a network analysis with model-based perturbation simulations

**DOI:** 10.3389/fpsyt.2026.1994414

**Published:** 2026-10-21

**Authors:** Xiaoyan Qiu, Xiaoqun Liu, Yumeng Miao, Pousheng Jiang, Ruixiang Gao

**Affiliations:** 1 Department of Psychology, School of Humanities and Management, Hunan University of Chinese Medicine, Changsha, China; 2 Xiangya School of Public Health, Central South University, Changsha, China; 3 School of Politics and Public Administration, Hunan Normal University, Changsha, China; 4 School of Psychology, South China Normal University, Guangzhou, China; 5 Center for Studies of Psychological Application, South China Normal University, Guangzhou, China; 6 Guangdong Key Laboratory of Mental Health and Cognitive Science, South China Normal University, Guangzhou, China; 7 Key Laboratory of Brain, Cognition and Education Sciences (South China Normal University), Ministry of Education, Guangzhou, China

**Keywords:** adolescents, bullying, victimization, perpetration, bystanders, network analysis, simulated intervention

## Abstract

**Background:**

School bullying threatens school safety and adolescent health. Research often treats victimization, perpetration, and bystander responses as distinct forms of involvement, leaving their within-person co-occurrence poorly understood.

**Methods:**

Using data from 7,913 students, this study applied network analysis and Node Identification and Response Analysis (NIRA) to examine behavioral co-occurrence and conditional associations across these dimensions and identify candidatenodes for future experimental validation.

**Results:**

Co-occurrence of all three dimensions exceeded that expected under independence (3.16%; O/E = 4.08, 95% CI [3.75, 4.43]), and formed an interconnected network with multiple cross-dimensional associations. Same-form victimization and perpetration, including verbal teasing, were positively connected, as were pro-bully bystander responses—particularly joining in bullying—and perpetration. Reverse-coded defender behaviors, including failure to intervene or report to adults, had limited direct connections with perpetration but yielded the largest projected changes in NIRA. In the primary NIRA analyses that excluded the perturbed node from the outcome, simulated mitigation of five bystander response nodes was associated with significant projected changes in the remaining 25-item aggregate score (Δ = 0.15–0.54; all ps_FDR < .001). BYS1 (failure to intervene) and BYS2 (failure to report) showed the largest projected changes under simulated threshold mitigation, with differences of −0.54 and −0.43 points, respectively.

**Conclusion:**

These exploratory simulations generate hypotheses that targeting reverse-coded defender bystander responses and promoting active intervention and adult reporting may warrant further investigation in future intervention studies. However, causality and real-world effectiveness remain to be further validated.

## 1 Introduction

In recent years, school bullying has become a major public health concern and has drawn increasing attention from educators, parents, and psychologists. A global report from the United Nations Educational, Scientific and Cultural Organization indicated that approximately one-third of students had experienced peer bullying during the previous month (1). This prevalence suggested that bullying was not an isolated event affecting only a small minority of students, but rather a school-related risk with global implications. Bullying victimization has been closely associated with depressive symptoms, self-devaluation, academic difficulties, and poor social adjustment among adolescents (2–4). More importantly, a growing body of evidence has suggested that bullying victimization and bullying perpetration may co-occur or develop in parallel. Adolescents who perpetrated bullying could also experience victimization, while those who were victimized could engage in bullying perpetration (5–7). Bystander responses, even among adolescents not directly involved in a bullying incident, may also be associated with a greater likelihood of perpetrating or experiencing bullying. These findings point to a potentially shared structure underlying the three forms of bullying involvement (8). However, this structure remains poorly understood.

Previous research recognized that individuals involved in bullying incidents could occupy different roles. The participant role approach views school bullying as a social process collectively enacted and sustained by the peer group. In addition to perpetrators and victims, it identified assistants, reinforcers, defenders, and outsiders as distinct bystander response roles (9). This approach positioned bystander responses as an integral part of bullying involvement rather than as a peripheral feature of the incident. Subsequent research, however, suggested that adolescents may not be confined to a single role. Students who experienced bullying could also exhibit a greater tendency to perpetrate bullying themselves. The between-person role distinctions described by the participant role approach could therefore coexist within the same individual as heterogeneous but interrelated behavioral tendencies. This pattern has been described as the “overlap between school bullying perpetration and victimization” (10, 11).

General strain theory attributed this overlap partly to individuals’ responses to strain. Bullying victimization represented a salient interpersonal stressor that could trigger anger, shame, and frustration. When adolescents lacked effective emotion regulation and self-control capacities, they might respond to such strain through retaliation, aggression, or bullying perpetration (12, 13). Alternatively, subcultural theories offered a different explanation centered on the surrounding cultural environment. Adolescents who remained embedded in social groups that endorsed violence and bullying could face pressure to perpetrate bullying to gain recognition or acceptance. Such behavior could also increase their exposure to aggression from other group members, making them more likely to experience bullying victimization themselves (14, 15).

Combining participant role approach with the evidence of victimization–perpetration overlap, this study further incorporated bystander responses to bullying into the co-occurrence structure of bullying perpetration and victimization. As an indirect form of bullying involvement, bystander responses could be closely related to adolescents’ direct experiences of perpetrating or experiencing bullying (8). Pro-bully reactions, such as joining in, assisting or reinforcing bullying, resembled active bullying perpetration in behavioral content. They could also share psychosocial foundations involving strain-related coping, peer-group norms, moral disengagement, and the justification of aggression (16–18). Chen et al. (19), for example, identified a subgroup of bullying-involved adolescents who simultaneously displayed follower and perpetrator tendencies. Furthermore, defending or avoidant reactions to witnessed bullying could likewise be related to empathy developed through prior victimization or concerns about retaliation (20). These considerations suggested that the conventional victimization–perpetration overlap framework should be extended by examining bullying perpetration, bullying victimization, and bystander responses to bullying within a common behavioral system.

Some previous studies examined the co-occurrence of bullying perpetration, victimization and bystander responses to bullying. However, they mainly focused on characterizing the profiles of those involved in bullying at a general level. Few studies investigated the conditional associations among the specific behavioral reactions underlying these three forms of involvement (21–23). Consequently, the behavioral basis for the co-occurrence of these three phenomena within the same individual remains unclear, making it difficult to implement targeted interventions to prevent or reduce bullying involvement among adolescents.

Network analysis offered a suitable framework for addressing this gap. It allowed specific behavioral reactions across bullying perpetration, bullying victimization, and bystander responses to bullying to be modeled within a single network. The resulting network estimated conditional associations that remained after all other behavioral reactions were controlled, making it possible to identify the specific behaviors through which the three forms of involvement co-occurred (24). Network centrality indices also helped characterize the relative structural position and embeddedness of each behavior. Nodes with higher centrality have often been treated as key candidate intervention targets (25, 26). However, centrality indices are typically calculated from the strength of connections between nodes and do not directly capture how changes in one node may affect the rest of the network. This limitation is particularly important in cross-sectional networks, where causal direction has not been established. A highly central node may therefore not represent the most effective intervention target (27).

Node Identification and Response Analysis (NIRA) provided a simulation-based alternative. The algorithm systematically altered the threshold parameter of each node and simulated the resulting changes in overall network activation. This procedure allowed the projected intervention effects of different behavioral nodes to be compared (28). NIRA has been increasingly applied to identify potential prevention and intervention targets in research on anxiety–depression comorbidity, trauma-related symptoms, and other forms of psychopathology (28–30). By combining network analysis with NIRA, the present study sought to characterize the co-occurrence structure of bullying perpetration, bullying victimization, and bystander responses to bullying in greater detail and to identify nodes that showed the largest projected changes under simulated threshold perturbation, as candidates for future validation.

Existing research indicated that adolescents could simultaneously exhibit tendencies toward bullying perpetration, victimization and bystander responses to bullying. However, understanding of the specific structural relationships between these three, as well as of candidate intervention hypotheses targeting this co-occurrence pattern, remains relatively limited. The present study used network analysis to examine their conditional associations and behavioral basis of co-occurrence at the level of specific behavioral reactions. NIRA was also applied to simulate interventions targeting different reactions involved in bullying and to identify nodes that, under simulated perturbation, showed the largest projected changes in overall network activation, as candidates for future validation. The study aimed to deepen the understanding of adolescents’ multidimensional involvement in bullying from a behavioral co-occurrence perspective. It also sought to provide exploratory evidence to inform hypotheses for future prevention research.

## 2 Materials and methods

### 2.1 Participants and procedure

The data were derived from a survey conducted between April and July 2018. Participants were recruited from a province in south-central China using a multistage stratified cluster sampling design. First, all prefecture-level cities in the province were divided into four geographic regions: eastern, western, southern, and northern. One prefecture-level city was randomly selected from each region as a sample city. Schools in each sample city were then stratified by urban–rural location, and four urban schools and four rural schools were randomly selected. Subsequently, six classes were randomly selected from each school, and all students in the selected classes were invited to participate. 9,156 students were invited to take part in the survey.

A total of 8,439 students completed the questionnaire, yielding a response rate of 92.2%. The questionnaires were screened using the following exclusion criteria: (1) missing key variables; (2) extensive blank responses; and (3) obvious logical inconsistencies (e.g., reporting “I have not been bullied” yet scoring high on bullying victimization items). After excluding questionnaires 7,913 participants were retained for the final analyses. The valid questionnaire rate was 93.8%. Among these, 3,766 were male (47.6%) and 4,147 were female (52.4%); 4,208 students (53.2%) attended rural schools, while 3,705 (46.8%) attended urban schools. In addition, there were 4,178 junior high school students (52.8%) and 3,735 senior high school students (47.2%). It should be noted that, due to privacy policies, the original survey did not collect students’ specific age information, and the current de-identified dataset does not contain an age variable. Therefore, age statistics are not reported in this study. The study was approved by the Human Research Ethics Committee of Hunan University of Chinese Medicine (approval no. HN-LL-SZR-2024-081). Informed consent was obtained from all participating students and their parents or legal guardians before data collection.

Because the data had a hierarchical structure, with students nested within classrooms and classrooms nested within schools, we fitted unconditional three-level random-intercept models for victimization, perpetration, and bystander responses. We calculated the intraclass correlation coefficients (ICC) at the classroom and school levels, with the results presented in **Table 1**. The classroom-level ICC were 0.049 for victimization, 0.143 for perpetration, and 0.025 for bystander responses, indicating relatively more pronounced classroom clustering for perpetration. School-level clustering was limited across all three dimensions. Because the primary aim of this study was to examine individual-level conditional associations among specific bullying behaviors, rather than mean differences or cross-level effects at the school or classroom levels, we used a standard single-level Ising model as the primary analysis. Considering that bullying is usually infrequent and concentrated in behaviors among a few students and their specific interactions, even if the overall class atmosphere is poor, it does not necessarily result in uniform bullying involvement across the entire class, we did not estimate a multilevel Ising network. The variance-component analysis was conducted solely to quantify the extent of clustering and inform the interpretation of the individual-level network results.

**Table 1:** Three-level variance components and intraclass correlation coefficients.

Outcome	N	School-level variance	Classroom-level variance	Residual variance	School-level ICC	Classroom-level ICC
Victimization	7913	0.0079	0.0097	0.1811	0.0399	0.0487
Perpetration	7913	0.0007	0.0159	0.0949	0.0059	0.1430
Bystander responses	7913	0.0050	0.0059	0.2243	0.0212	0.0251

### 2.2 Measures

Bullying involvement was assessed using the revised Olweus Bully/Victim Questionnaire (OBVQ) (31, 32). The questionnaire comprised two subscales assessing bullying perpetration and bullying victimization among secondary school students. The Chinese version has been widely used in research on school bullying among Chinese adolescents and has demonstrated satisfactory reliability and validity (32).

#### 2.2.1 Bullying perpetration

Bullying perpetration was assessed using nine items that asked students how often they had engaged in different forms of bullying during the current semester. One example item was, “I gave some classmates insulting nicknames, called them names, or teased and ridiculed them.” All items were rated on a 5-point scale: 1 = “never,” 2 = “once or twice,” 3 = “two or three times a month,” 4 = “about once a week,” and 5 = “several times a week.” Following the established OBVQ frequency criterion, responses of “never” and “once or twice” were coded as 0, whereas responses of “two or three times a month” or more frequently were coded as 1 (33). These values indicated whether each form of bullying perpetration fell below or met the criterion for repeated occurrence, respectively. In the present study, the subscale showed excellent internal consistency (Cronbach’s α = .94).

#### 2.2.2 Bullying victimization

Bullying victimization was also assessed using nine items that asked students how often they had experienced different forms of bullying at school during the current semester. One example item was, “Other students gave me insulting nicknames, called me names, or teased and ridiculed me.” The first seven items assessed traditional forms of bullying: verbal nickname teasing, social exclusion, physical attack, rumor spreading, extortion/property damage, forced tasks, and accent discrimination. The final two items assessed cyber verbal attack and cyber privacy violation. All items were rated on the same 5-point scale: 1 = “never,” 2 = “once or twice,” 3 = “two or three times a month,” 4 = “about once a week,” and 5 = “several times a week.” Responses of “never” and “once or twice” were coded as 0, whereas responses of “two or three times a month” or more frequently were coded as 1. These values indicated whether the frequency of each form of bullying victimization fell below or met the criterion for repeated occurrence, respectively. The subscale showed excellent internal consistency in the present study (Cronbach’s α = .97).

#### 2.2.3 Bullying bystander responses

Bystander responses were assessed using the Student Bystander Behavior Scale (SBBS) (34), an eight-item measure assessing how frequently students respond in different ways when witnessing a peer bullying another student. The eight reactions were intervene to stop, report to adults, pretend not seeing, leave situation, join bullying, assist bully, watch passively, and cheer/ridicule victim. Items 1–2 assessed defender behavior, Items 3–4 assessed outsider behavior, and the final four items assessed pro-bully behavior. Each item was rated on a 5-point scale ranging from 1 (“never”) to 5 (“always”). To ensure consistent node directionality, the intervene to stop and report to adults items were reverse-dichotomized. Responses of 1 (“never”) or 2 (“rarely”) were coded as 1, indicating failure to intervene to stop or failure to report to adults, whereas responses of 3 (“sometimes”) or above were coded as 0. For the remaining items, responses of 1 (“never”) or 2 (“rarely”) were coded as 0, and responses of 3 (“sometimes”) or above were coded as 1. The first two nodes represented reverse-coded defender responses, with higher scores indicating failure to intervene and failure to report bullying to adults, respectively. The next two nodes represented outsider responses, including pretending not to see the incident and leaving the situation, whereas the final four represented pro-bully bystander responses. In the present study, Cronbach’s α coefficients for the defender behavior, outsider behavior, and pro-bully behavior subscales were .68, .71 and .77, respectively, indicating generally acceptable internal consistency.

### 2.3 Data analysis

All analyses were conducted using R 4.6.0. First, the observed-to-expected ratio (O/E) was used to describe the co-occurrence of bullying victimization, bullying perpetration, and bystander responses. Following a 0/1 standard, the three dimensions were coded as “present” or “absent.” For example, a student was classified as “victimization–perpetration co-occurrence” when at least one item in both the bullying victimization and bullying perpetration dimensions was coded as 1, whereas all bystander items were coded as 0. Combining the three binary dimensions yielded eight possible patterns. Assuming that the three dimensions were mutually independent, the expected frequency of each pattern was calculated from their marginal probabilities. The 95% confidence intervals for the O/E ratios were estimated using bootstrap resampling with 5,000 resamples. An O/E ratio greater than 1 indicated that the corresponding pattern occurred more frequently than expected under the assumption of independence (35).

Since all variables in the network were binary, this study employed the Ising model to estimate the conditional association network among bullying victimization, bullying perpetration, and bystander. The network was estimated using the enhanced least absolute shrinkage and selection operator (eLasso) implemented in the IsingFit package. For each node, this method fitted a logistic regression model in which all other nodes served as predictors. The optimal regularization parameter was selected by minimizing the extended Bayesian information criterion (EBIC), resulting in a sparse network structure. The resulting undirected network consisted of an edge-weight matrix and a threshold vector (36). The network was visualized using the qgraph package.

Three centrality indices were subsequently calculated: Strength, Betweenness, and Expected Influence. Strength was defined as the sum of the absolute weights of all edges connected to a given node. Expected Influence retained the signs of the edge weights and was therefore more suitable for characterizing networks containing both positive and negative edges (25). The accuracy of the estimated edge weights was evaluated by calculating their 95% confidence intervals using 1,000 nonparametric bootstrap samples in the bootnet package. The stability of the centrality indices was assessed using correlation stability coefficients (CS coefficients) obtained from 1,000 case-dropping bootstrap samples (37).

Model-based threshold perturbation simulations were conducted using Node Identification and Response Analysis (NIRA), implemented in the nodeIdentifyR package. First, an unperturbed baseline response sample was generated from the estimated Ising model parameters. The threshold parameter of each node was then shifted by 2 standard deviations. Specifically, we applied a downward threshold shift, which mathematically increases the modeled probability that the corresponding node would be activated. This direction was used to represent the activation-increasing condition specified in the simulation framework. Each node was perturbed separately, while the threshold parameters of all other nodes were held constant. For each node-specific perturbation, a corresponding response sample was generated using the simulateResponses() function, with the sample size set equal to that of the baseline sample.

Finally, to quantify the projected change associated with perturbing each node while distinguishing the contribution of the perturbed node from changes in the remaining network, we defined two types of outcomes.

1. Leave-one-node-out aggregate score (primary outcome).

The perturbed node was excluded from both the unperturbed baseline sample and the corresponding perturbed sample. The aggregate score was then calculated across the remaining 25 nodes. This score served as the primary indicator of model-implied changes in the remaining network.

2. Role-specific subscale scores.

Separate scores were calculated for victimization (VIC1–VIC9; possible range: 0–9), perpetration (PER1–PER9; possible range: 0–9), and bystander responses (BYS1–BYS8; possible range: 0–8).

For each outcome, scores in the unperturbed baseline sample were compared with scores in each node-specific perturbed sample using independent-samples t tests. To account for multiple comparisons, p values were adjusted using the Benjamini–Hochberg false discovery rate (FDR) procedure. A *p_FDR* value < .05 was interpreted as evidence of a statistically detectable difference between the unperturbed and perturbed model-generated samples for the corresponding outcome (27).

## 3 Results

### 3.1 Observed and expected frequencies of bullying involvement patterns

**Table 2** presents the preliminary descriptive results for the co-occurrence patterns of bullying victimization, bullying perpetration, and bystander responses at a single time point. Among the 7,913 participants, bystander responses only was the most common pattern (66.17%), followed by none of the three dimensions present (14.60%) and victimization–bystander co-occurrence (11.94%). Compared with the frequency expected under the assumption that the three dimensions were mutually independent, co-occurrence of all three dimensions showed the greatest degree of clustering. Its observed frequency was approximately four times the expected frequency [3.16%; O/E = 4.08, 95% CI (3.75, 4.43)]. Although victimization–perpetration co-occurrence accounted for only a small proportion of the sample (0.40%), it also occurred significantly more often than expected under independence [O/E = 2.51, 95% CI (1.70, 3.35)]. In contrast, the victimization only, perpetration only, victimization–bystander co-occurrence, and perpetration–bystander co-occurrence patterns occurred less frequently than expected (O/E = 0.40–0.88). These findings provided preliminary evidence that the three dimensions of bullying involvement may co-occur within individual adolescents.

**Table 2:** Observed and expected frequencies of co-occurrence patterns across bullying victimization, bullying perpetration, and bystander.

Co-occurrence pattern	Observed n	Total sample (%)	Expected n under independence	O/E [95% CI]
None of the three dimensions present	1,155	14.6	1,065.0	1.08 [1.06, 1.11]
Victimization only	152	1.92	224.8	0.68 [0.58, 0.77]
Perpetration only	24	0.30	60.4	0.40 [0.25, 0.56]
Bystander responses only	5,236	66.17	5,118.0	1.02 [1.02, 1.03]
Victimization–perpetration co-occurrence	32	0.40	12.8	2.51 [1.70, 3.35]
Victimization–bystander co-occurrence	945	11.94	1,080.2	0.88 [0.85, 0.90]
Perpetration–bystander co-occurrence	119	1.50	290.5	0.41 [0.35, 0.47]
Co-occurrence of all three dimensions	250	3.16	61.3	4.08 [3.75, 4.43]
Victimization–pro-bully responses co-occurrences	957	12.1	929.7	1.03 [1.00, 1.06]
Perpetration–pro-bully responses co-occurrence	336	4.25	286.5	1.17 [1.12, 1.23]
Victimization–outsider responses co-occurrence	676	8.54	657.1	1.03 [0.98, 1.08]
Perpetration–outsider responses co-occurrence	228	2.88	202.5	1.13 [1.03, 1.22]
Victimization–reverse-coded defender responses co-occurrence	852	10.77	866.6	0.98 [0.95, 1.02]
Perpetration–reverse-coded defender responses co-occurrence	216	2.73	267.1	0.81 [0.74, 0.88]

Because bystander reactions may reflect distinct behavioral tendencies, we further examined the co-occurrence of victimization, perpetration, and specific bystander response dimensions (see **Table 2**). Perpetration co-occurred more frequently than expected with pro-bullying responses [O/E = 1.17, 95% CI (1.12, 1.23)] and outsider responses [O/E = 1.13, 95% CI (1.03, 1.22)], but less frequently than expected with reverse-coded defender responses [O/E = 0.81, 95% CI (0.74, 0.88)]. In contrast, victimization was associated with all three types of bystander responses at roughly expected levels. These findings suggest that different response types exhibit distinct co-occurrence patterns.

### 3.2 Ising network of adolescent bullying victimization, bullying perpetration, and bystander responses

Using the Ising model to estimate the network structure of symptoms associated with adolescent bullying involvement, the model comprises a total of 26 binary nodes. In total, 75 nonzero edges were identified in the estimated network, including 73 positive and 2 negative edges, corresponding to a network density of 0.231. 14 edges connected nodes from different communities, with the strongest cross-community edge observed between VIC1 and PER1 (*weight* = 1.96, all 14 cross-community edges are reported in **Supplementary Table S4**). The results of the network visualization and centrality analysis are shown in **Figures 1**, **2**.

**Figure 1:**
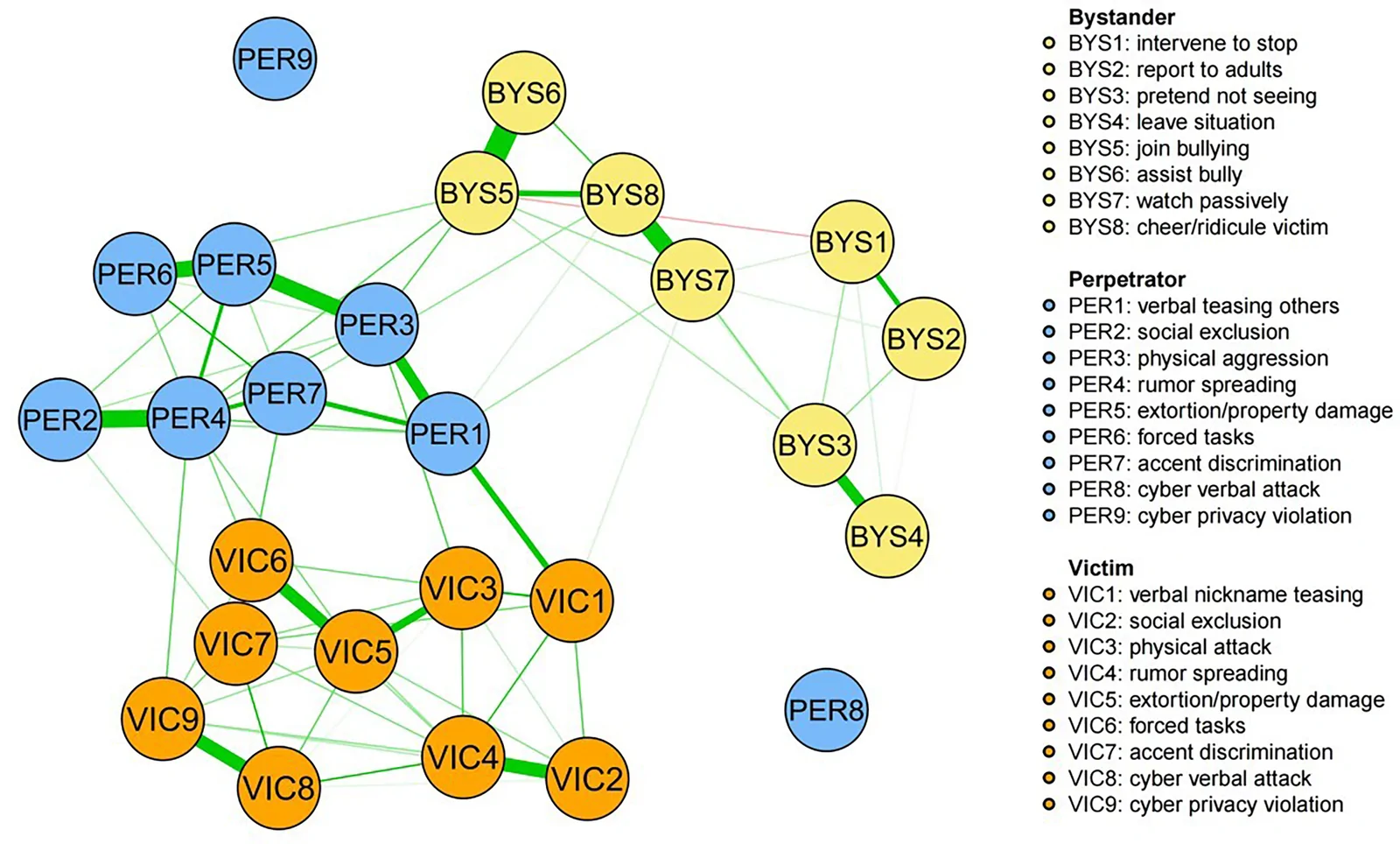
Ising network of bullying victimization, perpetration, and bystander responses. Green and red edges indicate positive and negative associations, respectively. Thicker and more saturated edges represent stronger associations. The absence of an edge indicates that the corresponding association was estimated as zero after eLasso regularization. BYS1 and BYS2 represent the reverse-coded items "intervene to stop" and "report to adults," respectively.

**Figure 2:**
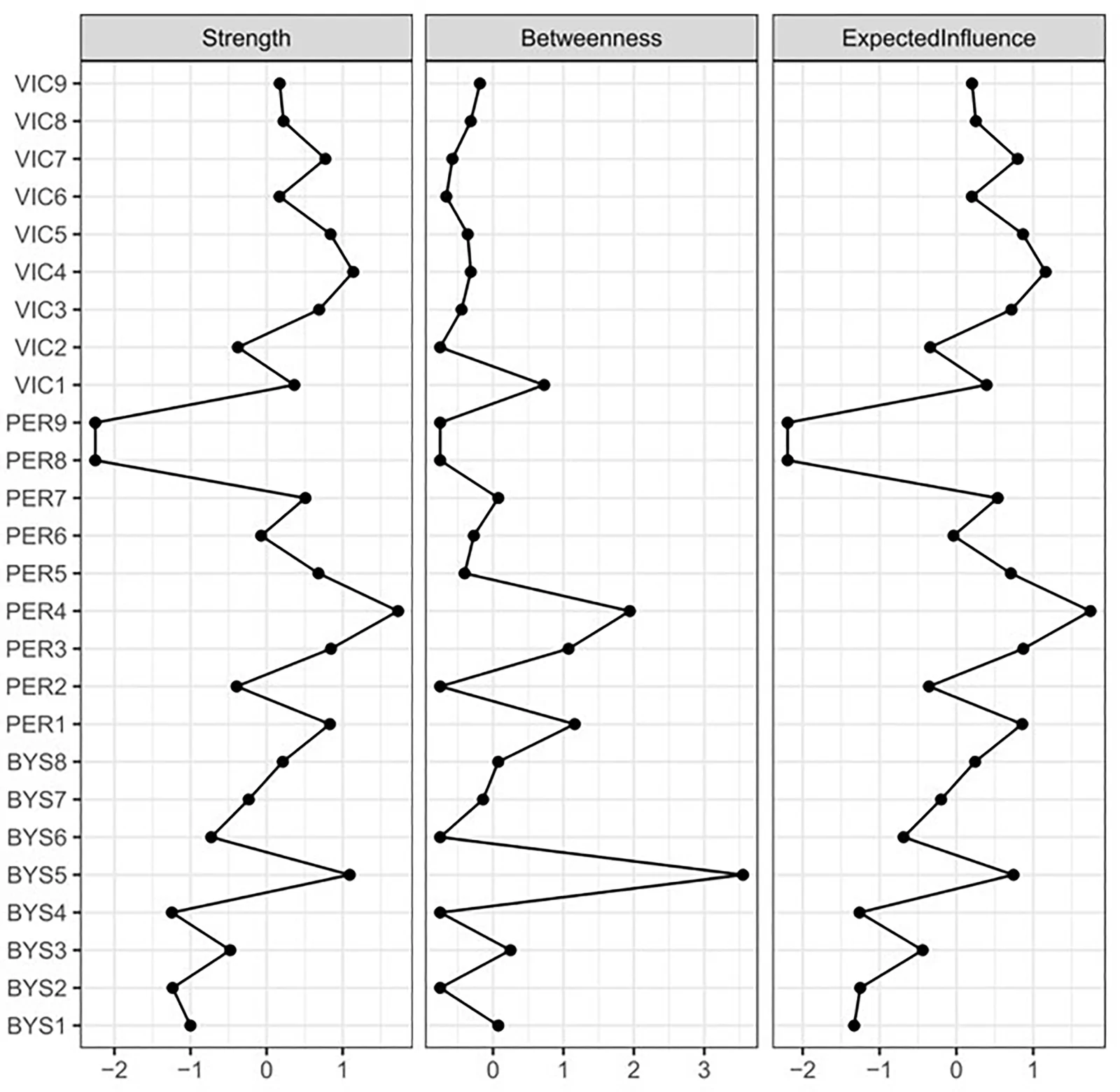
Strength, betweenness and expected influence of each node in the bullying network. The image shows the standardized centrality scores.

The network edge weight matrix shows that PER8 (online verbal abuse) and PER9 (online privacy infringement) have extremely low prevalence rates (1.0% and 0.8%, respectively), resulting in them having no connections to any other nodes and becoming isolated nodes in the network. The node with the highest strength is PER4 (spreading rumors, 11.40), followed by VIC4 (spreading rumors, 9.72), BYS5 (participating in bullying, 9.58), PER3 (physical aggression, 8.88), and PER1 (verbal teasing, 8.84). The nodes with the lowest strength (excluding outliers) are BYS4 (leaving the scene, 2.89), BYS2 (failure to report to adults, reverse-coded from “report to adults”, 2.91), and BYS1 (failure to intervene, reverse-coded from “intervene to stop”, 3.58), indicating that these bystander behaviors occupy relatively peripheral positions within the network. Node rankings based on Expected Influence were similar to those based on Strength (see **Supplementary Table S1** for the detailed centrality results).

The accuracy of the edge-weight estimates was assessed using nonparametric bootstrapping with 1,000 resamples. Most edge weights had relatively narrow 95% confidence intervals, indicating adequate estimation accuracy and stability (see **Supplementary Figures S1**, **2**). The stability of the centrality indices was assessed using case-dropping bootstrapping with 1,000 resamples. Strength showed acceptable stability, whereas betweenness showed relatively low stability and therefore it is not reported in the primary outcomes. It should be careful when explaining betweenness centrality should be interpreted cautiously (see **Supplementary Figure S3**).

Since the main network was estimated with an Ising model on dichotomized indicators, the original ordinal information may have been lost. To test robustness, we fitted a regularized Gaussian graphical model (EBICglasso) using the original ordinal variables (**Figure 3**). The strongest cross-dimensional edge was between VIC1 (verbal nickname teasing) and PER1 (verbal teasing; *weight* = 0.288), linking victimization and perpetration of the same behavioral form. PER4 and VIC4 also showed relatively high strength centrality.

**Figure 3:**
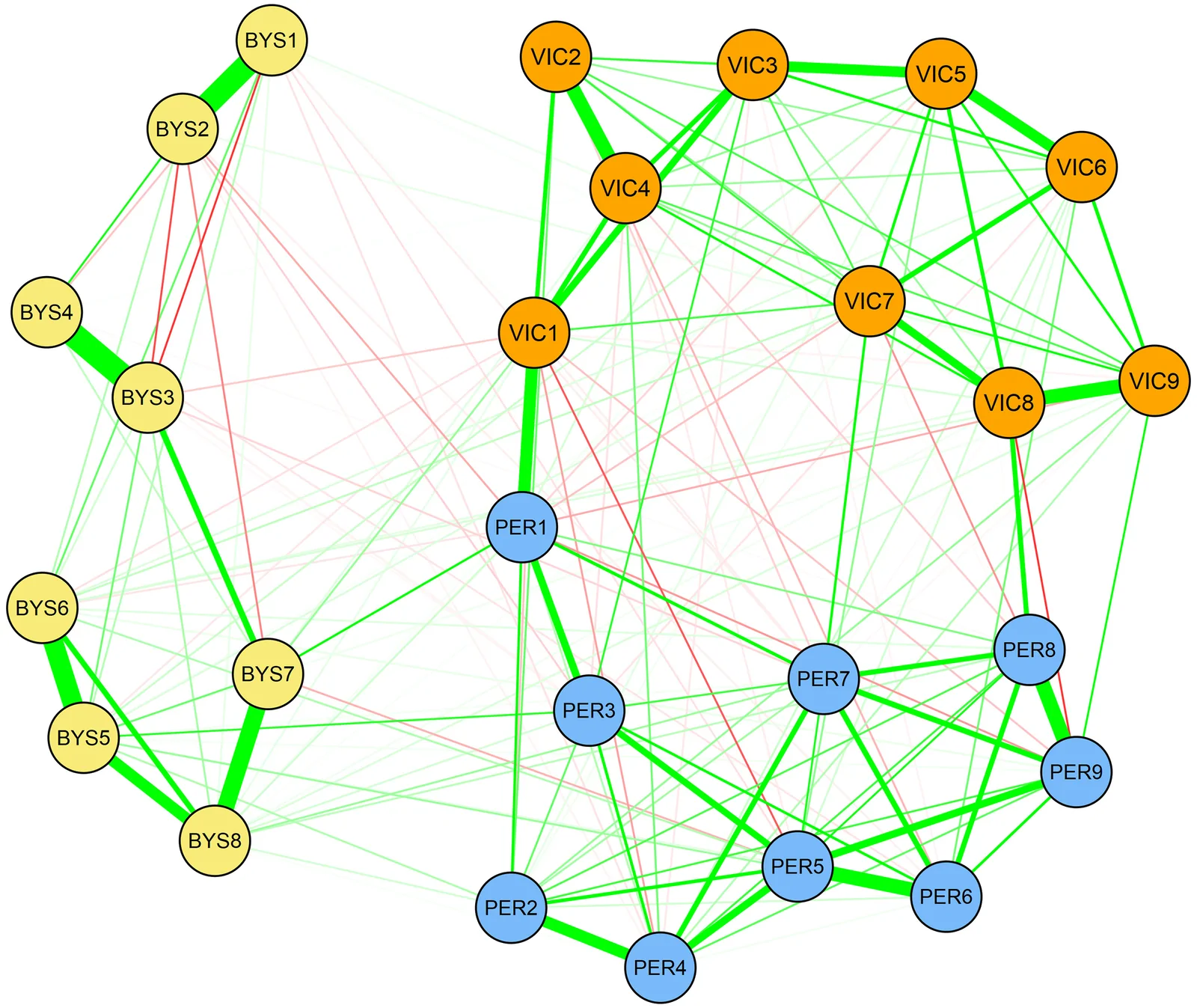
Gaussian graphical model network of the original bullying involvement nodes. EBICglasso was applied to the original five-point ordinal variables. Orange, blue, and yellow nodes stand for victimization, perpetration, and bystander responses. Green and red edges show positive and negative conditional associations, and the thickness of the edges reflects their absolute strength. BYS1 and BYS2 represent the reverse-coded items "intervene to stop" and "report to adults," respectively.

However, the findings for PER8 and PER9 differed across the two network specifications. In the Ising network, PER8 and PER9 were estimated as isolated nodes, whereas in the GGM they showed clear conditional associations with other nodes. The strongest association involving these nodes was the edge between PER8 and PER9 (*weight* = 0.333). One possible explanation is that dichotomization coded many adolescents with mild cyberbullying-perpetration experiences as zero, resulting in very sparse positive categories for PER8 and PER9. Under these conditions, the eLasso procedure may have shrunk weak associations to zero. This finding suggests that the isolated status of PER8 and PER9 was sensitive to the coding specification and should not be interpreted as evidence of behavioral independence. By retaining the ordinal variation in the original responses, the GGM may have captured conditional associations that were not apparent in the binary network.

### 3.3 Model-based threshold perturbation simulations

To further explore candidate nodes for future validation, we conducted model-based threshold perturbation simulations using the Node Identification and Response Analysis (NIRA) algorithm.

To further examine which node-specific threshold perturbations were associated with the largest projected changes in the remaining network, we applied the Node Identification and Response Analysis (NIRA) algorithm.

1. Changes in the aggregate score excluding the perturbed node

**Figure 4** presents the changes in the aggregate score of the remaining nodes after excluding the node subjected to perturbation. Among the 26 nodes, five node-specific threshold perturbations were associated with statistically detectable reductions in the aggregate score across the remaining 25 nodes after FDR correction (*p_FDR* < .05). All five nodes were bystander-response nodes (see **Table 3**).

**Figure 4:**
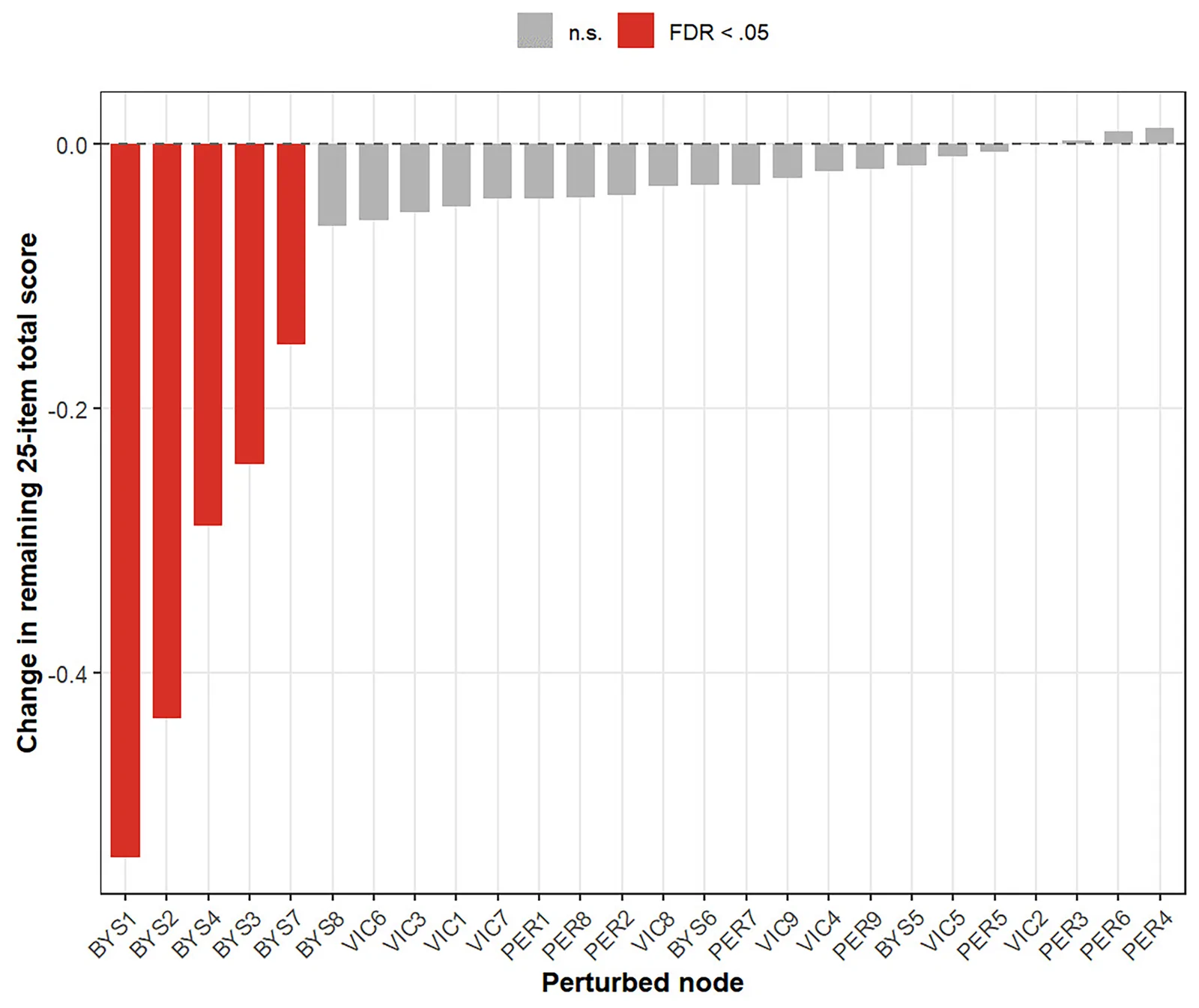
Model-implied changes in the remaining 25-item aggregate score under node-specific threshold perturbations, with the perturbed node excluded from the outcome. Red bars indicate statistically significant changes after FDR correction, whereas gray bars indicate nonsignificant changes. Negative values represent lower aggregate scores in the perturbed model-generated sample than in the baseline sample. These values reflect model-implied changes and should not be interpreted as causal effects of real-world interventions.

**Table 3:** Nodes showing significant simulated perturbation effects after excluding the perturbed node.

Node	Original mean	Intervention mean	Change	*t*	*p_FDR*
BYS1	2.11	1.57	-0.54	20.10	<.001
BYS2	2.17	1.73	-0.43	16.43	<.001
BYS4	2.55	2.26	-0.29	11.85	<.001
BYS3	2.72	2.48	-0.24	9.75	<.001
BYS7	2.81	2.65	-0.15	5.89	<.001

Independent-samples *t* tests compared the total network scores of the baseline sample with those of each simulated intervention sample after excluding the perturbed node. Positive *t* values indicate lower total network scores following simulated perturbation.

Specifically, the perturbation associated with BYS1 showed the largest projected change: the aggregate score decreased from 2.11 to 1.57, corresponding to a change of −0.54 (*t* = 20.10, *p_FDR* < .001). The corresponding changes for BYS2, BYS4, BYS3, and BYS7 were −0.43 (from 2.17 to 1.73; *t* = 16.43, *p_FDR* < .001), −0.29 (from 2.55 to 2.26; *t* = 11.85, *p_FDR* < .001), −0.24 (from 2.72 to 2.48; *t* = 9.75, *p_FDR* < .001), and −0.15 (from 2.81 to 2.65; *t* = 5.89, *p_FDR* < .001), respectively. The remaining 21 node-specific threshold perturbations did not produce statistically detectable differences in the aggregate score of the remaining nodes. Complete results are provided in **Supplementary Table S2**.

2. Role-Specific Subscale Analyses

**Table 4** summarizes model-implied changes in the role-specific subscales following node-specific threshold perturbations. Changes associated with bystander-node perturbations were largely confined to the bystander-response subscale. For example, perturbing BYS1 yielded a projected difference of −0.53 points in the sum score of the remaining seven BYS items (*p_FDR* < .001), whereas the VIC and PER subscales changed minimally (−0.01, *p_FDR* = .48; +0.001, *p_FDR* = .94). BYS2, BYS3, BYS4, and BYS7 showed comparable patterns. Simulated mitigation of VIC1 was associated with a projected difference of −0.04 points in the sum score of the remaining VIC items (*p_FDR* < .001), and several other VIC nodes showed changes within the VIC subscale. No perturbation of perpetration nodes significantly changed the remaining PER items. Detailed results are provided in **Supplementary Table S3**.

**Table 4:** Summary of model-implied changes in role-specific subscale scores after excluding the perturbed node.

Node	Change in VIC	*p_FDR*	Change in PER	*p_FDR*	Change in BYS	*p_FDR*
BYS1	-0.01	.48	+0.001	.94	-0.53	<.001
BYS2	-0.03	.05	+0.005	.94	-0.41	<.001
BYS4	-0.01	.44	+0.007	.82	-0.28	<.001
BYS3	-0.01	.60	+0.001	.94	-0.24	<.001
BYS7	-0.02	.08	-0.002	.94	-0.13	<.001
BYS8	-0.01	.53	+0.005	.82	-0.06	.066
VIC1	-0.04	<.001	-0.008	.97	-0.0002	.99
VIC3	-0.03	.03	+0.003	.97	-0.03	.89
VIC4	-0.04	.001	+0.005	.97	+0.01	.89
VIC6	-0.02	.15	+0.001	.97	-0.04	.47
PER1	-0.03	.05	+0.002	.97	-0.02	.89
PER2	-0.03	.05	-0.001	.97	-0.01	.89
PER3	-0.01	.32	+0.003	.97	+0.01	.89
PER4	-0.01	.53	+0.004	.97	+0.02	.89

VIC, PER, and BYS denote the victimization, perpetration, and bystander-response subscales, respectively. When the perturbed node belonged to a given subscale, that node was excluded from the corresponding subscale score. Changes represent differences between the perturbed and baseline model-generated samples.

3. Full-Network Aggregate Score Analysis

**Figure 5** presents the full-network aggregate score analysis, which included the perturbed node itself. Eleven nodes—VIC1, VIC3, VIC6, PER1, BYS1, BYS2, BYS3, BYS4, BYS6, BYS7, and BYS8—showed statistically significant model-implied changes after FDR correction (*p_FDR* < .05). However, this analysis could not distinguish the direct contribution of the perturbed node from changes in the remaining network. It was therefore treated as a supplementary analysis, and the primary conclusions were based on the analysis excluding the perturbed node.

**Figure 5:**
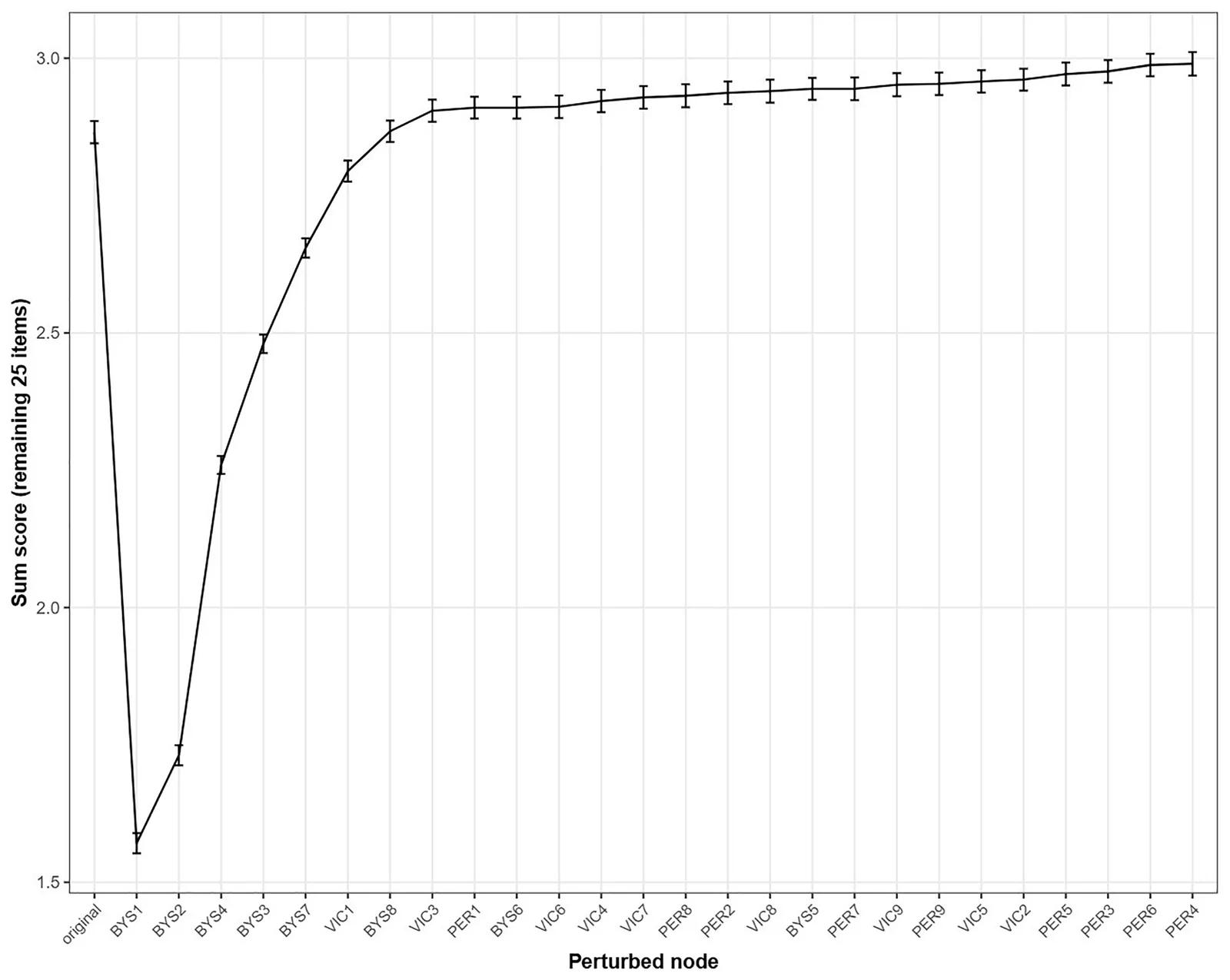
Projected changes under simulated threshold perturbations in the estimated Ising network. The network sum scores are represented by black dots, while the corresponding 95% confidence intervals are depicted by lines. The nodes are displayed according to the magnitude of the projected changes in the total network score, along with the original sum score before simulated perturbation.

## 4 Discussion

In school bullying incidents, the impact of bystander behavior on the phenomenon of bullying cannot be overlooked. The present study incorporated three distinct yet closely related dimensions of bullying involvement—bullying victimization, bullying perpetration, and bystander—into a single network system. NIRA was further used to identify behavioral nodes that showed relatively large projected changes under model-based threshold perturbations; these nodes may serve as candidates for future empirical validation, with bystander responses included in the network. Several findings are particularly noteworthy.

### 4.1 Co-occurrence network comprising victimization, perpetration, and bystander responses

Network analysis showed that bullying victimization, bullying perpetration, and bystander did not constitute three isolated behavioral dimensions among adolescents. Instead, they formed a cross-sectionally interconnected network of bullying involvement. Specifically, bullying perpetration showed meaningful positive conditional associations with specific experiences of both bullying victimization and bystander. For example, verbal teasing others was strongly connected to verbal nickname teasing. The perpetration dimension was also connected to the bystander-response dimension through pro-bullying responses such as join bullying. Many studies have documented substantial overlap between bullying perpetration and victimization. Some adolescents reported both experiencing and engaging in the same forms of bullying, particularly verbal, physical, and relational bullying. Such overlap may reflect hostile attribution processes or retaliatory responses to previous victimization (38–40).

Notably, in the estimated Ising network, pro-bullying bystander responses, including join bullying, watch passively, and cheer/ridicule victim, tended to show positive conditional associations with bullying perpetration. In contrast, reverse-coded defender responses (failure to intervene or report to adults) and outsider responses (pretending not to see the incident or leaving the situation) showed no clear associations with perpetration behaviors. Drawing on the participant role approach, Hutchings et al. (41) found substantial empirical overlap between assistants, reinforcers, and perpetrators. One possible explanation involves the diffusion of responsibility, which represents a mechanism of moral disengagement. Pro- bully bystander responses may reduce adolescents’ sense of personal responsibility and thereby weaken their moral agency (42, 43). Consequently, an increase in such experiences is closely associated with individuals more frequently assisting in or directly participating in bullying (44). Adolescents may therefore simultaneously display behavioral tendencies associated with multiple participant roles, including perpetrators, victims, and assistants. The possible reciprocal relationships and transitions among these roles warrant further discussion through longitudinal research designs.

### 4.2 Reverse-coded defender bystander responses as candidate nodes in a model-based perturbation analysis

The NIRA results revealed a noteworthy discrepancy: reverse-code defender and outsider bystander response nodes with relatively low centrality in the estimated Ising network produced the largest projected network-wide changes under simulated perturbations. Specifically, reverse-coded defender and outsider behavior —including failure to engage in intervention to stop or report to adults, pretend not seeing, and leave situation—exhibited weak direct connections with bullying perpetration and relatively low centrality. However, the largest reductions in the total network score occurred when the reverse-coded tendencies not to intervene and not to report were mitigated. These simulated changes represented encouraging students to intervene when witnessing bullying or to report the incident to adults. This finding indicated that the nodes with the widest connectivity in the network did not align with those expected to yield the greatest overall change following simulated modifications.

From the perspective of multidimensional bullying involvement within individuals, pro-bully bystander responses, such as join bullying, assist bully, and cheer/ridicule victim, shared active behavioral components with direct bullying perpetration. Their greater similarity in behavioral content may have made them more likely to form strong direct conditional associations in the co-occurrence network (45, 46). In contrast, reverse-coded defender and outsider responses reflected withdrawal from bullying situations and shared less behavioral content with other forms of bullying involvement. This distinction may help explain their relatively weak direct connections with other nodes (47).

This pattern did not mean that reverse-coded defender and outsider bystander responses were unimportant intervention targets. By contrast, these simulation findings suggest that perturbing reverse-coded defender-response nodes—for example, by promoting safe intervention and reporting to adults—may be associated with larger projected changes in the bystander-response subnetwork than mitigating individual aggressive behavior. In the simulated model, mitigating pro-bully responses did not produce comparably large projected reductions in the overall network. It may, in theory, lead to passive bystanding without fostering genuine moral engagement; this remains to be tested (48).

Social cognitive theory distinguishes between two levels of moral agency: inhibitory and proactive. The former manifests as self-restraint to avoid committing or supporting harmful acts, while the latter manifests as assuming moral responsibility and proactively taking actions that benefit others (49). Compared with simply discouraging problematic behavior, promoting active intervention and reporting may strengthen broader psychological resources, including moral awareness, personal responsibility, and defender self-efficacy (34, 50). Within the same individual, these resources may reduce passive or pro-bully responses when witnessing bullying. They may also increase adolescents’ willingness to seek adult support when victimized and strengthen their ability to resist peer pressure to participate in bullying (51–53). Thus, although these nodes lacked extensive direct connections, perturbing them produced relatively large changes in the overall simulated perturbation network. Nevertheless, because the NIRA results were derived from simulated interventions based on cross-sectional network parameters, the actual effectiveness of potential intervention targets requires further evaluation using experimental or quasi-experimental designs.

### 4.3 Exploratory implications for future intervention

The findings offer exploratory implications for future intervention research forward addressing adolescents’ involvement in school bullying, particularly the co-occurrence and potential mutual reinforcement of different forms of bullying involvement. First, victimization, perpetration, and bystander responses may be better regarded as interrelated dimensions of bullying involvement rather than as mutually exclusive roles. The present findings indicate that perpetration co-occurs with both victimization and bystander responses. Accordingly, future anti-bullying interventions might need to consider not only controlling perpetrators’ behavior and protecting victims but also providing psychological support and moral education to reduce the potential for different dimensions of bullying involvement to reinforce one another (54).

Second, future school-based intervention research may consider defender bystander responses as candidate targets. Bystander intervention training may strengthen students’ willingness and ability to take safe protective action when witnessing bullying, and whether such training is associated with changes in other bullying-related behaviors remains to be tested (55).

Finally, beyond overt physical aggression and violent conflict, future prevention efforts may consider monitoring the emergence and spread of relational aggression, particularly rumor spreading, which occupied a highly central position in the co-occurrence network. Such behavior is often difficult to detect promptly but can spread rapidly and draw additional bystanders into circulating harmful information, potentially escalating an interpersonal incident into group-based bullying. Therefore, improving the identification of and response to rumor spreading and other less overt forms of bullying may be a candidate component of future school-based anti-bullying efforts (56).

### 4.4 Limitations and future directions

This study has several limitations. First, its cross-sectional design means the estimated network reflects conditional associations among bullying perpetration, victimization, and bystander responses, rather than dynamic processes or directional relationships. The findings should therefore not be interpreted as evidence of causal effects or transitions between bullying roles. Future multi-wave longitudinal or multilevel temporal network studies are needed to examine temporal relationships among individuals’ behavioral reactions during specific bullying incidents (57).

Second, the study relied primarily on student self-reports, which may be affected by recall bias, social desirability, and common method bias. In particular, socially disapproved behaviors may have been underreported. Future research should combine self-reports with peer nominations, teacher observations, and anonymous incident records to cross-validate findings across sources (58).

Third, NIRA identifies nodes that produce relatively large simulated effects under a specified network structure and parameter-perturbation rule; it does not establish causally effective intervention targets that have been validated in real-world settings. The model-based prominence of “failure to intervene” and “failure to report to adults” therefore requires further examination through class-level randomized interventions or quasi-experimental studies. Such studies should also evaluate the durability and feasibility of intervention effects, as well as potential risks, including peer retaliation against students who report bullying (59).

In addition, although we quantified school- and classroom-level clustering using variance-component models, the primary single-level Ising network did not explicitly partition within-classroom, between-classroom, and between-school associations. Future studies should apply multilevel network models and cluster-aware resampling procedures to examine whether the observed edge structure and centrality patterns remain after accounting for the hierarchical data structure.

Finally, the candidate behaviors identified in this study were not externally replicated or evaluated using longitudinal, experimental, or quasi-experimental designs. Therefore, the model-based projections should be interpreted as hypothesis-generating findings rather than evidence of causal intervention effects.

## 5 Conclusion

Using network analysis and NIRA at the level of specific behavioral reactions, this study examined the co-occurrence of adolescents’ bullying victimization, perpetration, and bystander responses, as well as candidate nodes for future validation. The findings showed that these three forms of bullying involvement were not isolated within individuals but were interconnected through multiple conditional associations, together constituting a co-occurrence network of bullying involvement. Specifically, bullying perpetration was associated with both victimization and pro-bullying bystander responses. NIRA further indicated that reverse-code defender and outsider bystander responses, despite their relatively low centrality, showed considerable projected changes under simulated threshold perturbation. Simulated mitigation of “failure to intervene” and “failure to report” produced the largest projected reductions in the aggregate score of the remaining 25 items. However, role-specific analyses indicated that these changes were largely confined to the remaining bystander-response items, with no significant changes in the victimization or perpetration subscales. Thus, the results suggest that bystander-response nodes may represent candidate leverage points for changing related bystander behaviors, rather than established targets for reducing victimization or perpetration. These findings generate hypotheses that future school-based interventions might consider not only perpetrators and victims separately but also attend to the clustering of multiple forms of bullying involvement. Facilitating such a shift may represent a candidate hypothesis for future anti-bullying intervention research.

## Data Availability

The raw data supporting the conclusions of this article will be made available by the authors, without undue reservation.
