# Supplementary material for: The co-occurrence of victimization, perpetration, and bystander responses in adolescent school bullying: a network analysis with model-based perturbation simulations
Source: Front Psychiatry. 2026 Oct 21;17:1994414. doi: 10.3389/fpsyt.2026.1994414 (PMC13732128; doi:10.3389/fpsyt.2026.1994414)
Supplement: Supplementary file 1 [file SupplementaryFile1.docx]

Supplementary Materials

This supplementary file contains the supplementary tables and figures referenced in the manuscript. Table and figure numbering follow the manuscript text.

**Supplementary Table 1.** Raw Centrality Indices for Nodes in the Adolescent Bullying Ising Network

| **Dimension** | **Nodes** | **Strength** | **Betweenness** | **Expected Influence** |
| --- | --- | --- | --- | --- |
| Vicitim | VIC1 | 7.50 | 68 | 7.50 |
|  | VIC2 | 5.37 | 0 | 5.37 |
|  | VIC3 | 8.43 | 14 | 8.43 |
|  | VIC4 | 9.72 | 20 | 9.72 |
|  | VIC5 | 8.86 | 18 | 8.86 |
|  | VIC6 | 6.94 | 4 | 6.94 |
|  | VIC7 | 8.66 | 8 | 8.66 |
|  | VIC8 | 7.09 | 20 | 7.09 |
|  | VIC9 | 6.94 | 26 | 6.94 |
| Perpetrator | PER1 | 8.84 | 88 | 8.84 |
|  | PER2 | 5.32 | 0 | 5.32 |
|  | PER3 | 8.88 | 84 | 8.88 |
|  | PER4 | 11.40 | 124 | 11.40 |
|  | PER5 | 8.41 | 16 | 8.41 |
|  | PER6 | 6.25 | 22 | 6.25 |
|  | PER7 | 7.91 | 38 | 7.91 |
|  | PER8 | 0.00 | 0 | 0.00 |
|  | PER9 | 0.00 | 0 | 0.00 |
| Bystander | BYS1 | 3.58 | 38 | 2.51 |
|  | BYS2 | 2.91 | 0 | 2.74 |
|  | BYS3 | 5.08 | 46 | 5.08 |
|  | BYS4 | 2.89 | 0 | 2.71 |
|  | BYS5 | 9.58 | 198 | 8.51 |
|  | BYS6 | 4.37 | 0 | 4.37 |
|  | BYS7 | 5.78 | 28 | 5.78 |
|  | BYS8 | 7.06 | 38 | 7.06 |

**Supplementary Table 2.** Model-implied changes in the remaining 25-item aggregate score following node-specific threshold perturbations, with the perturbed node excluded

| **Node** | **Baseline mean (excluding target)** | **Perturbed mean (excluding target)** | **Change (perturbed - baseline)** | ***t*** | ***p*** | ***FDR-adjusted p*** |
| --- | --- | --- | --- | --- | --- | --- |
| BYS1 | 2.110 | 1.571 | －0.539 | 20.103 | 0.000 | 0.000 |
| BYS2 | 2.166 | 1.731 | －0.435 | 16.435 | 0.000 | 0.000 |
| BYS4 | 2.548 | 2.260 | －0.289 | 11.850 | 0.000 | 0.000 |
| BYS3 | 2.723 | 2.480 | －0.243 | 9.752 | 0.000 | 0.000 |
| BYS7 | 2.807 | 2.655 | －0.152 | 5.893 | 0.000 | 0.000 |
| BYS8 | 2.929 | 2.867 | －0.062 | 2.220 | 0.026 | 0.115 |
| VIC6 | 2.969 | 2.912 | －0.058 | 1.974 | 0.048 | 0.180 |
| VIC3 | 2.957 | 2.904 | －0.052 | 1.814 | 0.070 | 0.227 |
| VIC1 | 2.843 | 2.795 | －0.048 | 1.740 | 0.082 | 0.237 |
| VIC7 | 2.971 | 2.929 | －0.042 | 1.438 | 0.150 | 0.355 |
| PER1 | 2.952 | 2.910 | －0.042 | 1.459 | 0.145 | 0.355 |
| PER8 | 2.972 | 2.932 | －0.041 | 1.374 | 0.169 | 0.362 |
| PER2 | 2.976 | 2.937 | －0.039 | 1.337 | 0.181 | 0.362 |
| VIC8 | 2.972 | 2.940 | －0.032 | 1.096 | 0.273 | 0.473 |
| BYS6 | 2.941 | 2.910 | －0.031 | 1.091 | 0.275 | 0.473 |
| PER7 | 2.975 | 2.944 | －0.031 | 1.055 | 0.291 | 0.473 |
| VIC9 | 2.978 | 2.952 | －0.026 | 0.879 | 0.379 | 0.580 |
| VIC4 | 2.943 | 2.922 | －0.021 | 0.737 | 0.461 | 0.666 |
| PER9 | 2.972 | 2.953 | －0.019 | 0.650 | 0.516 | 0.706 |
| BYS5 | 2.961 | 2.944 | －0.016 | 0.572 | 0.567 | 0.737 |
| VIC5 | 2.968 | 2.958 | －0.010 | 0.345 | 0.730 | 0.840 |
| PER5 | 2.977 | 2.971 | －0.006 | 0.210 | 0.834 | 0.903 |
| VIC2 | 2.960 | 2.961 | 0.001 | －0.028 | 0.978 | 0.978 |
| PER3 | 2.974 | 2.976 | 0.002 | －0.082 | 0.935 | 0.972 |
| PER6 | 2.978 | 2.988 | 0.010 | －0.328 | 0.743 | 0.840 |
| PER4 | 2.978 | 2.990 | 0.012 | －0.403 | 0.687 | 0.840 |

Note. Both the unperturbed baseline and each node-specific perturbed sample exclude the current target node. The score is calculated across the remaining 25 nodes.

**Supplementary Table 3.** Model-implied changes in role-specific subscale total scores following node-specific threshold perturbations

| **Node** | **VIC change** | ***FDR-adjusted p*** | **PER change** | ***FDR-adjusted p*** | **BYS change** | ***FDR-adjusted p*** |
| --- | --- | --- | --- | --- | --- | --- |
| VIC1 | -0.039 | 0.000 | -0.008 | 0.824 | 0.000 | 0.994 |
| VIC2 | -0.022 | 0.003 | 0.001 | 0.941 | 0.022 | 0.889 |
| VIC3 | -0.030 | 0.000 | 0.003 | 0.941 | -0.026 | 0.831 |
| VIC4 | -0.039 | 0.000 | 0.005 | 0.824 | 0.012 | 0.889 |
| VIC5 | -0.023 | 0.009 | 0.001 | 0.941 | 0.012 | 0.889 |
| VIC6 | -0.019 | 0.029 | 0.001 | 0.941 | -0.039 | 0.413 |
| VIC7 | -0.035 | 0.001 | 0.000 | 0.941 | -0.008 | 0.889 |
| VIC8 | -0.008 | 0.270 | 0.003 | 0.941 | -0.027 | 0.831 |
| VIC9 | -0.029 | 0.025 | 0.001 | 0.941 | 0.002 | 0.970 |
| PER1 | -0.026 | 0.058 | 0.002 | 0.000 | -0.018 | 0.889 |
| PER2 | -0.031 | 0.029 | -0.001 | 0.824 | -0.007 | 0.889 |
| PER3 | -0.014 | 0.306 | 0.003 | 0.941 | 0.014 | 0.889 |
| PER4 | -0.009 | 0.513 | 0.004 | 0.941 | 0.017 | 0.889 |
| PER5 | -0.017 | 0.234 | 0.008 | 0.824 | 0.003 | 0.970 |
| PER6 | -0.008 | 0.513 | 0.007 | 0.824 | 0.010 | 0.889 |
| PER7 | -0.031 | 0.025 | 0.007 | 0.941 | -0.007 | 0.889 |
| PER8 | -0.023 | 0.101 | 0.000 | 0.824 | -0.018 | 0.889 |
| PER9 | -0.028 | 0.046 | 0.002 | 0.824 | 0.007 | 0.889 |
| BYS1 | -0.010 | 0.482 | 0.001 | 0.941 | -0.531 | 0.000 |
| BYS2 | -0.027 | 0.049 | 0.005 | 0.824 | -0.414 | 0.000 |
| BYS3 | -0.006 | 0.597 | 0.001 | 0.941 | -0.237 | 0.000 |
| BYS4 | -0.011 | 0.441 | 0.007 | 0.824 | -0.284 | 0.000 |
| BYS5 | -0.022 | 0.101 | -0.001 | 0.941 | 0.007 | 0.889 |
| BYS6 | -0.018 | 0.206 | -0.002 | 0.941 | -0.011 | 0.209 |
| BYS7 | -0.025 | 0.067 | -0.002 | 0.941 | -0.125 | 0.000 |
| BYS8 | -0.009 | 0.513 | 0.005 | 0.824 | -0.058 | 0.000 |

*Note.* VIC, PER, and BYS denote the victimization, perpetration, and bystander-response subscales, respectively. Change values were calculated as the perturbed score minus the baseline score. The reported p values were adjusted using the Benjamini–Hochberg false discovery rate procedure.

**Table S4.** Cross-community edges in the estimated network

| **Node 1** | **Node 2** | **Community 1** | **Community 2** | ***Weight*** |
| --- | --- | --- | --- | --- |
| VIC1 | PER1 | Victimization | Perpetration | 1.96 |
| VIC7 | PER7 | Victimization | Perpetration | 1.188 |
| VIC3 | PER3 | Victimization | Perpetration | 1.174 |
| VIC9 | PER4 | Victimization | Perpetration | 1.103 |
| PER4 | BYS5 | Perpetration | Bystander | 1.073 |
| PER3 | BYS5 | Perpetration | Bystander | 1.055 |
| VIC6 | PER6 | Victimization | Perpetration | 0.897 |
| VIC5 | PER4 | Victimization | Perpetration | 0.856 |
| PER6 | BYS5 | Perpetration | Bystander | 0.745 |
| PER7 | BYS8 | Perpetration | Bystander | 0.647 |
| PER1 | BYS7 | Perpetration | Bystander | 0.566 |
| VIC7 | PER2 | Victimization | Perpetration | 0.512 |
| VIC1 | BYS7 | Victimization | Bystander | 0.251 |
| PER1 | BYS8 | Perpetration | Bystander | 0.25 |

*Note.* All 14 cross-community edges were positive. The strongest cross-community edge connected VIC1 and PER1 (weight = 1.960), followed by VIC7–PER7 (weight = 1.188) and VIC3–PER3 (weight = 1.174).

**Supplementary Figure 1**. Accuracy of Edge-Weight Estimates Based on Nonparametric Bootstrapping

*Note. Red points represent edge weights estimated from the original sample, black points represent bootstrap means, and gray horizontal lines represent 95% bootstrap confidence intervals. Edges are ordered according to their estimates in the original sample. Narrower confidence intervals indicate greater estimation accuracy.*

**Supplementary Figure 2**. Bootstrapped Difference Test for Estimated Edge Weights

*Note. Black cells indicate statistically significant differences between pairs of edge weights (p < .05), whereas gray cells indicate nonsignificant differences. Blue diagonal cells represent comparisons of each edge weight with itself.*

**Supplementary Figure 3.** Stability of Strength, Betweenness, and Expected Influence assessed using case-dropping bootstrapping
